# Perceptual Learning at Higher Trained Cutoff Spatial Frequencies Induces Larger Visual Improvements

**DOI:** 10.3389/fpsyg.2020.00265

**Published:** 2020-02-21

**Authors:** Di Wu, Pan Zhang, Chenxi Li, Na Liu, Wuli Jia, Ge Chen, Weicong Ren, Yuqi Sun, Wei Xiao

**Affiliations:** ^1^Department of Medical Psychology, Air Force Medical University, Xi’an, China; ^2^Department of Psychology, The Ohio State University, Columbus, OH, United States; ^3^School of Nursing, Yueyang Vocational Technical College, Yueyang, China; ^4^Department of Nursing, Air Force Medical University, Xi’an, China; ^5^Department of Psychology, School of Education Science, Huaiyin Normal University, Huai’an, China; ^6^School of Arts and Design, Zhengzhou University of Light Industry, Zhengzhou, China; ^7^Department of Psychology, Hebei Normal University, Shijiazhuang, China; ^8^Department of Systems Neuroscience, Universitätsklinikum Hamburg-Eppendorf, Hamburg, Germany

**Keywords:** perceptual learning, cutoff spatial frequency, visual acuity, contrast sensitivity function, visual improvement

## Abstract

It is well known that extensive practice of a perceptual task can improve visual performance, termed perceptual learning. The goal of the present study was to evaluate the dependency of visual improvements on the features of training stimuli (i.e., spatial frequency). Twenty-eight observers were divided into training and control groups. Visual acuity (VA) and contrast sensitivity function (CSF) were measured and compared before and after training. All observers in the training group were trained in a monocular grating detection task near their individual cutoff spatial frequencies. The results showed that perceptual learning induced significant visual improvement, which was dependent on the cutoff spatial frequency, with a greater improvement magnitude and transfer of perceptual learning observed for those trained with higher spatial frequencies. However, VA significantly improved following training but was not related to the cutoff spatial frequency. The results may broaden the understanding of the nature of the learning rule and the neural plasticity of different cortical areas.

## Introduction

During the last few decades, perceptual learning has been found to improve visual performance from simple visual feature discrimination to complex object recognition, such as contrast (Yu et al., 2004; Moret et al., 2018; Zhang et al., 2018), stimulus orientation (Wang et al., 2010; Jehee et al., 2012), motion (Ball and Sekuler, 1987; Larcombe et al., 2018), stereoacuity (Ding and Levi, 2011; Xi et al., 2014), vernier acuity (Fahle and Edelman, 1993; Hung and Seitz, 2014), shape (Gilbert and Sigman, 2000; Gilbert et al., 2009), and facial recognition (Gold et al., 1999). Previous studies have shown that the improvement in learning is highly specific to the characteristics of the trained task or stimulus (Fahle and Morgan, 1996; Li et al., 2004; Huang et al., 2007) or even to the trained eye or retinal location (Karni and Sagi, 1991). However, an increasing number of studies have identified the generalization of learning to other stimuli, tasks, and contexts (Jeter et al., 2010; McGovern et al., 2012). Task difficulty (Ahissar and Hochstein, 1997; Liu, 1999) or precision (Jeter et al., 2009) of the training and/or transfer tasks, training protocol (Xiao et al., 2008; Hung and Seitz, 2014; Huang et al., 2017), and the brain stimulation (Contemori et al., 2019) were found to influence the degree of transfer or specificity. This transference substantially broadens the value of the implications of perceptual learning.

Notably, perceptual learning has been found to have great potential to improve visual functions such as visual acuity (VA) and contrast sensitivity (CS) in individuals with amblyopia (Chung et al., 2006; Zhou et al., 2006; Campana et al., 2014; Barollo et al., 2017), myopia (Tan and Fong, 2008; Camilleri et al., 2014; Casco et al., 2014), presbyopia (Polat et al., 2012; Sterkin et al., 2018), central or peripheral vision loss (Casco et al., 2018; Zhang et al., 2009; Chung, 2011; Maniglia et al., 2016), dyslexia (Gori and Facoetti, 2014), and even normal vision (Deveau et al., 2014). For example, Huang et al. (2008) trained observers with amblyopia or normal vision in a grating detection task near each individual’s cutoff spatial frequency. For the amblyopic observers, training significantly improved VA in the amblyopic eye (2.7 dB). No significant VA improvement was observed in normal observers. Additionally, for amblyopic and normal observers, training at the trained spatial frequency induced significant improvements in CS by 10.7 and 5.6 dB, respectively. Therefore, perceptual learning has begun to be considered a non-invasive rehabilitation method for various clinical conditions (Lu et al., 2011).

A successful vision training protocol that is suitable for application must consider both effectiveness and cost (Lu et al., 2016). To obtain the greatest benefits from training, it is necessary to better classify the subtypes of the population using visual performance assessment batteries. An individual customized training procedure based on subgroups contributes to maximizing the training effect. Several previous studies using a grating detection task trained observers near each individual’s cutoff spatial frequency, which was defined as the spatial frequency at which the contrast threshold of the pretraining CS function (CSF) was a constant (Zhou et al., 2006, 2012; Huang et al., 2008; Yan et al., 2015; Zhang et al., 2018); that is, each observer was trained at individual spatial frequencies. Thus, an interesting question is who gains the most from perceptual learning. Do observers who were trained at a high cutoff spatial frequency gain greater visual improvement? Different spatial frequencies involve different cortical sites. Specifically, V1 neurons at the occipital pole (close to the skull) have higher preferred spatial frequencies than those located deeper within the calcarine sulcus (Horton, 2006; Henriksson et al., 2008; Yu et al., 2010). Therefore, the current study focused on the effect of trained cutoff spatial frequencies on visual improvement following perceptual learning, which also contributes to understanding the mechanisms underlying neural plasticity in different cortical areas.

The current study aimed to assess whether the gains following perceptual learning vary with the trained cutoff spatial frequencies. All observers were trained for eight daily sessions in a monocular grating detection task near their individual cutoff spatial frequencies. The same training protocol has been employed to train both normal and amblyopic populations and has been shown to be able to improve visual performance (Zhou et al., 2006, 2012; Huang et al., 2008). The cutoff spatial frequency was calculated as the spatial frequency at which the contrast threshold of pretraining CSF was 0.50. The current study provides empirical evidence regarding a customized training program for observers with different cutoff frequencies.

## Materials and Methods

### Observers

The participants consisted of 28 senior high school male students with cycloplegic spherical equivalence (SE) not exceeding ±0.5 diopter (D). Their VA on the Chinese Tumbling E Chart was better than 0.8 (20/25) (see Table 1 for details). None of the participants were aware of the purpose of the study. This study involving human participants was reviewed and approved in advance by the Research Ethics Committee at the Air Force Medical University and adhered to the principles of the Declaration of Helsinki. The participants and their legal guardians provided written informed consent for participation in this study.

**TABLE 1:** Observer characteristics.

					Acuity	Trained
Group	Sub	Symbol	Age	Eye	(logMAR)	SF (c/°)
Training	S1	*	18	R	–0.200	39.97
	S2	+	18	L	–0.297	39.17
	S3	○	17	R	–0.138	38.31
	S4	×	17	L	–0.150	36.94
	S5	♢	16	R	–0.297	36.57
	S6	□	17	L	–0.138	30.57
	S7	△	17	R	–0.175	28.23
	S8	▽	16	R	–0.050	25.27
	S9	▷	16	R	–0.150	24.48
	S10	◁	16	R	–0.088	19.48
	S11	✩	17	R	0.100	18.65
	S12	✡	16	R	0.013	16.95
	S13	▲	18	R	0.025	16.44
	S14	▼	18	R	0.100	14.84
	S15	▶	17	L	0.000	14.59
	S16	◀	17	L	–0.100	13.71
	S17	★	18	L	–0.075	11.62
	S18	•	17	L	0.025	9.39
Control	S19		17	R	–0.163	
	S20		18	R	–0.150	
	S21		17	L	–0.100	
	S22		17	L	–0.075	
	S23		16	L	–0.297	
	S24		17	L	–0.200	
	S25		18	R	–0.200	
	S26		17	R	–0.200	
	S27		17	L	–0.150	
	S28		16	L	–0.297	

### Apparatus

The study was performed on a computer running MATLAB programs with PsychToolbox extensions (Brainard, 1997). Stimuli were displayed on a gamma-corrected monitor with a spatial resolution of 1600 × 900 pixels, a refresh rate of 75 Hz, and a mean luminance of 35 cd/m^2^. An 8-bit screen was used in this study. To improve the measurement accuracy of low contrast stimuli, the Bit-Stealing technique was used to increase the number of levels of gray (Tyler et al., 1992). Observers placed their head on a chin rest and viewed the display monocularly in a dimly lit room, with an opaque patch on the other eye. The display subtended 5.56° × 3.13° at a viewing distance of 4.64 m. The entire experiment was performed without optical correction.

### Experimental Design

Visual acuity was measured with the Chinese Tumbling E Chart and converted to MAR acuity. All observers were randomly divided into training (S1–S18) and control groups (S19–S28). The eye with poorer VA served as the trained eye.

For the training group, the experiment contained three consecutive phases: (a) pretraining measurements of the monocular VA and CSF in the trained eye, (b) eight daily sessions of monocular training in the sine-wave grating detection task at each individual’s cutoff spatial frequency, and (c) post-training measurements that were the same as the pretraining measurements. To exclude the possibility that the visual improvements were only due to the repetition of the same VA and CSF tests at the pre- and post-training measurements, we collected a small sample of ten observers with the same criteria as the training group. All observers practiced approximately 50 trials before the pretraining measurements. Retention of the CSF and VA improvements was also assessed at least 5 months after training.

The CSF measure contained seven blocks of 100 trials and lasted approximately 45 min. CS was defined as the reciprocal of the contrast threshold for detecting a sine-wave grating with 79.3% accuracy at seven spatial frequencies of 1, 4, 8, 16, 24, 32, and 48 c/°. All spatial frequencies were interleaved among trials. The stimuli were vertical sinusoidal gratings of 2° in diameter with a half-Gaussian ramp (σ = 0.25).

The training phase contained eight daily sessions, each with seven blocks of 80 trials, and lasted approximately 35 min. Observers were trained at their individual cutoff spatial frequencies, defined as the spatial frequency at which the contrast threshold of the pretraining CSF was 0.50. Observers received 20¥ once the contrast threshold in the current session was lower than that in the last session; otherwise, there was no monetary reward.

### Procedure

A two-interval, forced-choice, sine-wave grating detection task was used for the assessment of the CSF and training. A target grating was randomly presented in one of two 100 ms temporal intervals that were signaled by a brief tone at the beginning of each and separated by 500 ms. Observers indicated the signal interval using the computer keyboard by pressing the left button when the signal was present in the first interval and pressing the right button otherwise. During training, a brief tone followed each correct response. During the CSF measurements, a brief tone followed each response regardless of its accuracy. The response also initiated the next trial.

Thresholds were assessed using an adaptive staircase method, termed a three-down/one-up staircase procedure (Levitt, 1971). This method, expected to asymptote at 79.3% correct, decreased signal contrast by 10% (multiplying the previous value by 0.9) after every three consecutive correct responses and increased signal contrast by 10% after every incorrect response. One hundred trials were used to estimate the contrast threshold for detecting a sine-wave grating at a particular spatial frequency. A reversal results when the staircase changes its direction (changing from increasing to decreasing contrast or vice versa). The first five (if the number of total reversals was odd) or four (if even) reversals were excluded. We averaged the contrasts of the remaining reversals to assess the contrast threshold for detecting the grating of a certain spatial frequency. Based on the results of the pilot testing, the starting contrast for each staircase was set close to the expected threshold.

### Data Analysis

Unless noted otherwise, the significance level was *p* < 0.05 throughout the paper. Pearson correlation with two-tailed probability values was used for all reported correlations.

The log CSF graphs log CS (1/threshold) as a function of spatial frequency. The area under the log CSF (AULCSF), which provides a broad measure of CS across all spatial frequencies, was calculated to evaluate the improvement (Koop et al., 1996).

For VA and CS, the magnitude of the improvements was calculated as follows:

(1) $$I_{\text{individual}}=20\,{\text{log}}_{10}\,\frac{\mathrm{posttraining}\,\mathrm{measure}}{\mathrm{pretraining}\,\mathrm{measure}}\,\mathrm{dB}$$

In the section “Results,” the average magnitude of improvements for group level is reported: *I*_group_ = ∑*I*_individual_/*N* (*N* is valid sample size). Group percent improvement can be calculated from the average dB improvement:

(2) $$P_{\text{group}}=\left(10^{\frac{I_{\text{group}}}{20}}-1\right)\times 100\%.$$

For VA and AULCSF, the retention of training effects was calculated by(VA_retest_−VA_pre_)/(VA_post_−VA_pre_)×100% and (AULCSF_retest_−AULCSF_pre_)/(AULCSF_post_−AULCSF_pre_)×100%, respectively.

**FIGURE 1:**
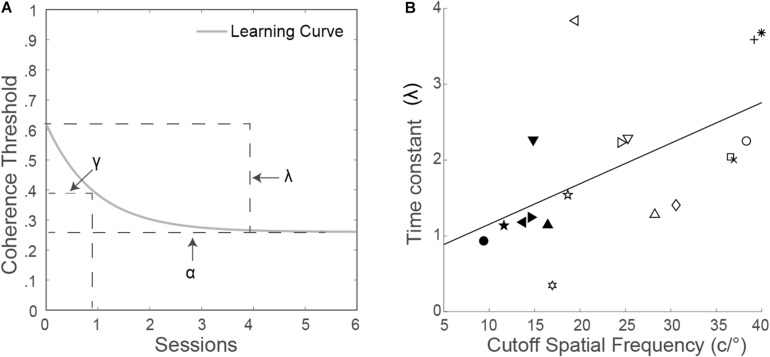
**(A)** Exponential function with the three parameters. **(B)** Time constant (γ) as a function of the cutoff spatial frequency. Each symbol represents an observer. The solid line is the best fitting regression line.

To obtain the learning curves, an exponential function was used (Zhang et al., 2019; Zhao et al., 2019):

(3) Contrast⁢threshold⁢(t)=α+λexp(-t/γ)

where α is the asymptotic performance level, λ is the dynamic range of learning, γ is the time constant of the exponential function, and *t* is the training session number. Figure 1A shows the exponential learning curve with the three parameters.

A non-linear least square method, implemented in MATLAB (MathWorks, Natick, MA, United States), was used to minimize the sum of squared differences between the model predictions and measured values. The goodness of fit was estimated by the following:

(4) $$r^{2}=1.0-\frac{\sum {\left(y_{\text{measured}}-y_{\text{predicted}}\right)}^{2}}{\sum {\left[y_{\text{measured}}-\text{mean}\,\left(y_{\text{measured}}\right)\right]}^{2}}$$

where *y*_*measured*_ and *y*_*predicted*_ represent the measured and predicted values, respectively. The mean(*y*_measured_) is the mean of all the measured values.

## Results

### The Time Constant to Plateau

The time constant γ was the time to reach asymptote, calculated by fitting the exponential function with the non-linear least square method. Ten contrast thresholds at trained cutoff spatial frequencies can be used to fit the function including eight training sessions and two testing sessions (Supplementary Figure S2). The time constant γ was positively correlated with the trained cutoff spatial frequency (*r* = 0.57, *p* = 0.014; Figure 1B).

### CSF Improvements

**FIGURE 2:**
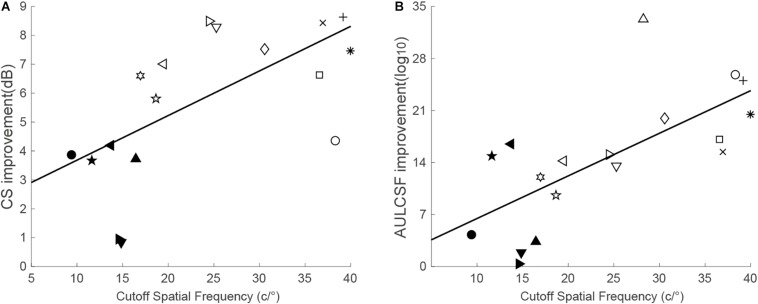
**(A)** CS improvement at the trained spatial frequency as a function of the cutoff spatial frequency. **(B)** AULCSF improvement as a function of the cutoff spatial frequency. The solid line is the best fitting regression line.

Based on the pre- and post-training CSF (Supplementary Figure S1), CS at the trained spatial frequency was calculated. Significant improvement in CS at the trained spatial frequency was found for the trained eye, *t*(17) = 11.61, *p* < 0.001, *d* = 2.74. Averaged across observers, the magnitude of improvement was 5.87 ± 0.61 dB (mean ± SE, range: 0.83–9.19 dB; or *P*_group_ = 96.56%). The improvement was significantly correlated with the cutoff spatial frequency (*r* = 0.63, *p* = 0.005), with higher gains observed for those with higher cutoff frequencies (Figure 2A).

Training at the cutoff spatial frequency improved CS over a range of spatial frequencies (Huang et al., 2008). Thus, AULCSF was calculated to evaluate the improvements across all spatial frequencies. Similarly, the post-training AULCSF was significantly larger than the pretraining AULCSF, *t*(17) = 7.12, *p* < 0.001, *d* = 1.68. The absolute improvements ranged from 0.36 to 33.29 log_10_ units (mean ± SE: 14.60 ± 2.05 log_10_ units). In addition, the improvement in AULCSF was significantly related to the cutoff spatial frequency (*r* = 0.69, *p* = 0.001), indicating that training in higher cutoff spatial frequencies generated greater AULCSF improvement (Figure 2B).

### Generalization of Perceptual Learning

**FIGURE 3:**
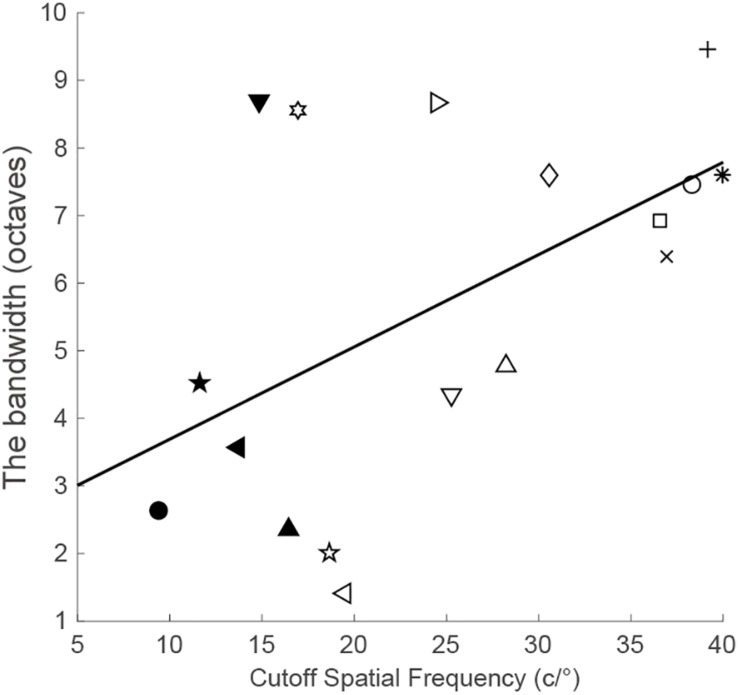
The bandwidth of perceptual learning as a function of the cutoff spatial frequency.

To quantify the generalizability to other untrained spatial frequencies, we computed the bandwidth of perceptual learning, that is, the bandwidth of the difference between the post-training and pre-training CSFs in the trained eye (Huang et al., 2008). Only observers with a significant amount of performance improvement at the training frequency were included in the results reported here. The correlation analysis showed that the bandwidth of perceptual learning was positively connected with the cutoff spatial frequency (*r* = 0.54, *p* = 0.025; Figure 3). This suggests that observers with higher spatial frequencies have broader bandwidths of perceptual learning. On average, the bandwidth was 5.70 ± 0.65 octaves (mean ± SE, range: 1.41–9.46 octaves).

### Visual Acuity

**FIGURE 4:**
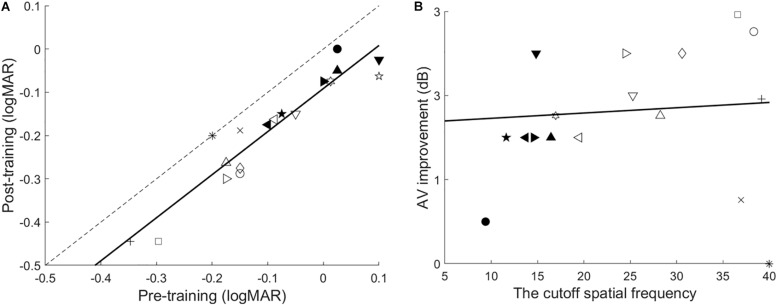
**(A)** The correlation of pre-training and post-training VA. The dashed line is the identity line with a slope of 1. The data points representing improved VA are located below this line. The solid line is the best-fitting linear model. **(B)** VA improvement as a function of the cutoff spatial frequency. The solid line is the best fitting regression line.

The paired *t*-test showed that training significantly improved VA, *t*(17) = 9.032, *p* < 0.001, *d* = 2.13. Furthermore, the best-fitting linear regression model was performed with the pre- and post-training VA of each observer. Almost all observers improved, as indicated by the clustering of data below the identity line. The model had a slope of 0.99, *r*^2^ = 0.89, *p* < 0.001. *F*-tests were conducted to compare the slope of the linear model and 1. If the linear model and the identity line were parallel, no effect of pretraining VA was found on the training improvement of VA. As shown in Figure 4A, no significant difference in the trained eye was found, *F*(1,32) = 0.007, *p* = 0.936.

Additionally, the magnitude of the VA improvements was not correlated with the cutoff spatial frequency (*r* = 0.08, *p* = 0.760), indicating that the trained cutoff frequency does not influence the VA gains (Figure 4B). The magnitude of improvement was 1.82 ± 0.20 dB (mean ± SE, range: 0–3.26 dB; or *P*_group_ = 23.31%).

### Retention

We remeasured the VA and CSF of the trained eye 5 months after training in 11 of the 18 observers. The observers retained 67.44% and 59.41% of their VA and AULCSF improvements, respectively. In addition, the trained cutoff spatial frequency was not correlated with the retention of training effects for VA (*r* = 0.41, *p* = 0.209) and AULCSF (*r* = 0.38, *p* = 0.256).

### Control Group

For the control group, there was no significant change in AULCSF improvements, *t*(9) = 0.955, *p* = 0.364, *d* = 0.30. The same pattern was found for VA; that is, no significant change in VA after training was found, *t*(9) = 0.599, *p* = 0.564, *d* = 0.19.

## Discussion

The current research aimed to investigate the effect of the participants’ individual cutoff spatial frequencies on the time constant for perceptual learning, magnitude, and transfer of perceptual learning after training in a monocular grating detection task. The results showed that perceptual learning induced significant visual improvements (i.e., CS at the cutoff spatial frequency, AULCSF, and VA), of which CS and AULCSF were closely related to the trained spatial frequency, with higher gains observed for those with higher spatial frequencies. In particular, after eight training sessions, the time constant to reach asymptote was positively related to the cutoff spatial frequency. A smaller time constant occurred at lower spatial frequencies. In contrast, the magnitude of CS improvement at higher spatial frequencies was larger than it was at lower spatial frequencies. Furthermore, the magnitude of AULCSF improvement at higher spatial frequencies was larger than that at lower spatial frequencies. However, the cutoff spatial frequency did not influence the VA improvements. Additionally, the bandwidth of perceptual learning was broader at higher cutoff spatial frequencies. Finally, the retention of VA and AULCSF improvements after 5 months was not related to the cutoff spatial frequency.

Overall, these results suggest that the magnitude and transfer of perceptual learning are tied to the trained cutoff spatial frequencies, with higher gains observed for higher cutoff spatial frequencies. However, the cutoff spatial frequency has no effect on improvements in VA and retention. These results may have important practical implications for the development of personalized training programs and theoretical importance for the understanding of neural plasticity in different cortical areas.

Contrast sensitivity function describes how visual sensitivity varies as a function of grating spatial frequency, providing a comprehensive measure of the visual system over a wide range of spatial frequencies in both normal and abnormal vision (Ginsburg, 2003). The CSF is closely correlated with daily visual functions, because our visual environment includes a variety of visual stimuli with a broad spatial frequency spectrum and a wide range of contrasts (Hou et al., 2015). Different spatial frequencies may involve various cortical areas. In a study using transcranial direct current stimulation (tDCS), the effects of tDCS were the largest for stimuli presented at higher spatial frequencies (Richard et al., 2015). The authors suggested that the effects of tDCS on CS were the greatest when higher spatial frequency gratings were used, as neurons with higher preferred spatial frequencies would be most influenced by tDCS. Since the magnitude of the electric field generated by tDCS is greater at the cortical surface, the superficial layers in the primary visual cortex near the apex of the calcarine sulcus contain neurons with higher preferred spatial frequencies than cells further from the apex (Horton, 2006; Henriksson et al., 2008; Yu et al., 2010). In the current study, observers with higher cutoff spatial frequencies generated larger magnitude and greater transfer of perceptual learning than those with lower cutoff spatial frequencies. That is, training at different spatial frequencies induced different levels of visual improvement, which is regarded as a manifestation of various degrees of cortical plasticity (Gilbert et al., 2001; Yotsumoto et al., 2008). Since various spatial frequencies recruit diverse cortical areas, it is plausible that the neural plasticity of diverse cortical areas is different. More specifically, V1 neurons at the occipital pole (close to the skull) are more plastic than those located deeper within the calcarine sulcus. Overall, the larger improvements and broader bandwidth, suggesting more plasticity in higher trained spatial frequencies, provides a strong empirical and theoretical basis for perceptual learning.

Previous evidence has also found that there was an asymmetric effect of training at high and low spatial frequencies (Huang et al., 2008). Specifically, training near the cutoff spreads broadly across lower spatial frequencies, whereas training at higher spatial frequencies spreads narrowly, which is opposite with our results. The possible reason is the differences in study samples. Huang et al. (2009) required the adults with normal or corrected-to-normal vision to train at higher spatial frequencies, and adults with unilateral anisometropic amblyopia to train at lower spatial frequencies. The results showed that the bandwidth of perceptual learning in the amblyopic visual system (lower trained spatial frequencies) is much broader than that in the normal visual system. However, all observers in the current study had normal or close to normal vision. Many previous studies found that neural plasticity is more robust in amblyopia (Huang et al., 2009; Astle et al., 2011). Thus, the neural plasticity of amblyopia may cover the plastic differences that result from various spatial frequencies.

Interestingly, our results showed that observers who gained more improvement had larger time constant. Li et al. (2008) found that those with mild amblyopia more quickly reach asymptote than those with severe amblyopia (5.55 and 18.75 h, respectively). Obviously, the amount of improvement is positively related to the time constants; that is, more time is needed to reach asymptote. In their study, individuals with deep amblyopia gained more improvement and thus required more time. If so, in current study, observers training at high spatial frequencies improved more, thus they need more time (larger exponential time constant) to reach plateau. The time constant to reach plateau was dependent on the amount of improvement, with a larger time constant observed for those with larger improvement.

Generally, the cutoff frequencies are positively related to the initial VA; that is, observers with high cutoff frequencies tend to have better pretraining VA. This pattern of results was also verified in current study (*p* < 0.001). Thus, our results may also suggest that the initial VA had a positive effect on visual improvement, which is opposite to previous studies. For example, Yan et al. (2015) found greater acuity improvement for observers with worse initial VA. Yehezkel et al. (2016) also found that subjects with more advanced presbyopia and thus poorer initial VA showed the greatest improvement in VA. Even so, there were at least two differences between these previous studies and this study. First, the initial vision and age differed. Yan et al. (2015) recruited myopic adult observers; Zhou et al. (2012) recruited presbyopic adults; and the present study recruited adolescent observers with normal or close to normal vision. Second, the incentive conditions were different. In contrast with the other two studies, the current study provided a high monetary reward once the participants’ performance was better than that in the last session. These factors may be the moderating variables that change the effect of initial performance on perceptual learning. The issue regarding the relationship between initial performance and improvement remains ambiguous. Specifically, patients with age-related macular degeneration learned at an equivalent rate to age-matched participants with normal vision (Astle et al., 2015). After equating the observers’ initial thresholds at the various eccentricities by spatially scaled stimuli, or introducing different levels of crowding, the original different amounts of improvement at each eccentricity disappeared (Astle et al., 2013). Thus, the effect of pretraining performance on perceptual learning must be deeply investigated by strictly controlling the experimental conditions in future research.

In contrast with the magnitude and transfer of perceptual learning, the trained cutoff frequency failed to relate to the VA improvement and retention of training effects. VA refers to the ability to discriminate two stimuli separated in space at high contrast relative to the background. It is different from CSF, which measures the CS in a wide range of spatial frequencies. Regarding retention, the results of this study suggest that the trained spatial frequency influences the training effect rather than the retention of the training effect.

Although we did not get the Bonferroni corrected/adjusted *p*-value for correlations in section “Result,” it is still meaningful to discuss this issue. Since the cutoff spatial frequency has been used in seven different correlations, the Bonferroni corrected *p* is 0.05/7 = 0.007. With this conservative criterion, the correlations between trained cutoff spatial frequency and time constant to plateau and the bandwidth become not significant. In other words, only the magnitude of perceptual learning is tied to the trained cutoff spatial frequencies. This may be due to the short training paradigm that a few subjects did not reach the plateau. On the one hand, the time constant to plateau and trained spatial frequencies was not correlated after Bonferroni correction. Based on the individuals’ learning curves, a few of observers did not reach their final plateau, especially those with higher cutoff spatial frequencies. But it does not influence our finding: observers who gained more improvement had larger time constant. If observers with higher cutoff frequencies achieved asymptotic performance with enough sessions, the time constant would be larger. On the other hand, the bandwidth of perceptual leaning did not relate to trained spatial frequencies. Based on the previous studies, the relationship between generalization and the amount of learning remains controversial (Lengyel and Fiser, 2019). Intuitively, generalization and learning should go hand in hand. However, excessive training could induce overfitting, which could weaken extra learning, but it also substantially decreases generalization. Indeed, several behavioral studies have confirmed this conjecture (Jeter et al., 2010; Hussain et al., 2012). Thus, the learning and generalization should be deeply investigated in future research.

The current study trained observers with a sine-wave grating detection task and found that the training effect was modulated by the trained spatial frequency. It is unclear whether the conclusions of the current study can be generalized to other tasks. For example, observers in a coherent motion detection task who trained at different coherence levels show a fixed percent correct. It has been demonstrated that various tasks recruit different brain areas. For example, the grating detection task used in the current study is related to the primary visual cortex (V1; Schoups et al., 2001; Ghose et al., 2002; Furmanski et al., 2004), while V4 is engaged in the orientation discrimination task (Yang and Maunsell, 2004). Thus, the generalizability of this study to other perceptual learning tasks regarding different brain areas should be further investigated.

From a practical standpoint, the present research indicates that it would be beneficial to translate perceptual learning from the laboratory to applications. Many studies have suggested that perceptual learning is a potential treatment for amblyopia, low myopia, presbyopia, and other issues. The ideal vision training would be able to obtain maximal gains with minimal costs. Redundant training wastes time and costs, and insufficient training does not produce the expected training effect. Thus, it is important to optimize vision training. The current study found that individual spatial frequencies have an effect on the magnitude of visual improvement, which provides empirical support for optimizing and customizing training programs for various observers. Additionally, the pretraining vision in this study was normal or close to normal. Regardless, the vision significantly improved after training, indicating perceptual learning can improve vision, which has important practical value for occupations that have special requirements for vision such as pilots. Additionally, this study has important theoretical implications for understanding the neural plasticity of different cortical areas. In the primary visual cortex, V1 neurons at the superficial layers near the apex of the calcarine sulcus have higher preferred spatial frequencies, inducing greater improvement after perceptual learning. Thus, this result provides insights into the neural mechanisms underlying the efficiency of perceptual learning.

In summary, we found that observers with higher cutoff spatial frequencies generated a higher magnitude and generalization of visual improvement than those with lower cutoff spatial frequencies after contrast detection training, even though all the observers had normal or close to normal vision. This finding suggests that the training stimulus is an important component in applications of perceptual learning. The development of a successful training protocol should consider individual differences in the training stimuli.

## Data Availability Statement

All datasets generated for this study are included in the article/Supplementary Material.

## Ethics Statement

The study involving human participants was reviewed and approved in advance by the Research Ethics Committee at the Air Force Medical University and adhered to the principles of the Declaration of Helsinki. The participants and their legal guardians provided written informed consent for participation in this study.

## Author Contributions

DW, PZ, GC, and NL performed the research. DW and PZ analyzed the data. DW and WJ wrote the manuscript. DW, PZ, CL, WR, and YS revised the manuscript and work on the review. PZ programmed the code. WX supervised the whole work.

## Conflict of Interest

The authors declare that the research was conducted in the absence of any commercial or financial relationships that could be construed as a potential conflict of interest.
