# Supplementary material for: Perceptual Learning at Higher Trained Cutoff Spatial Frequencies Induces Larger Visual Improvements
Source: Front Psychol. 2020 Feb 21;11:265. doi: 10.3389/fpsyg.2020.00265 (PMC7047335; doi:10.3389/fpsyg.2020.00265)
Supplement: Supplementary file 1 [file Data_Sheet_1.docx]

Supplementary Information

**
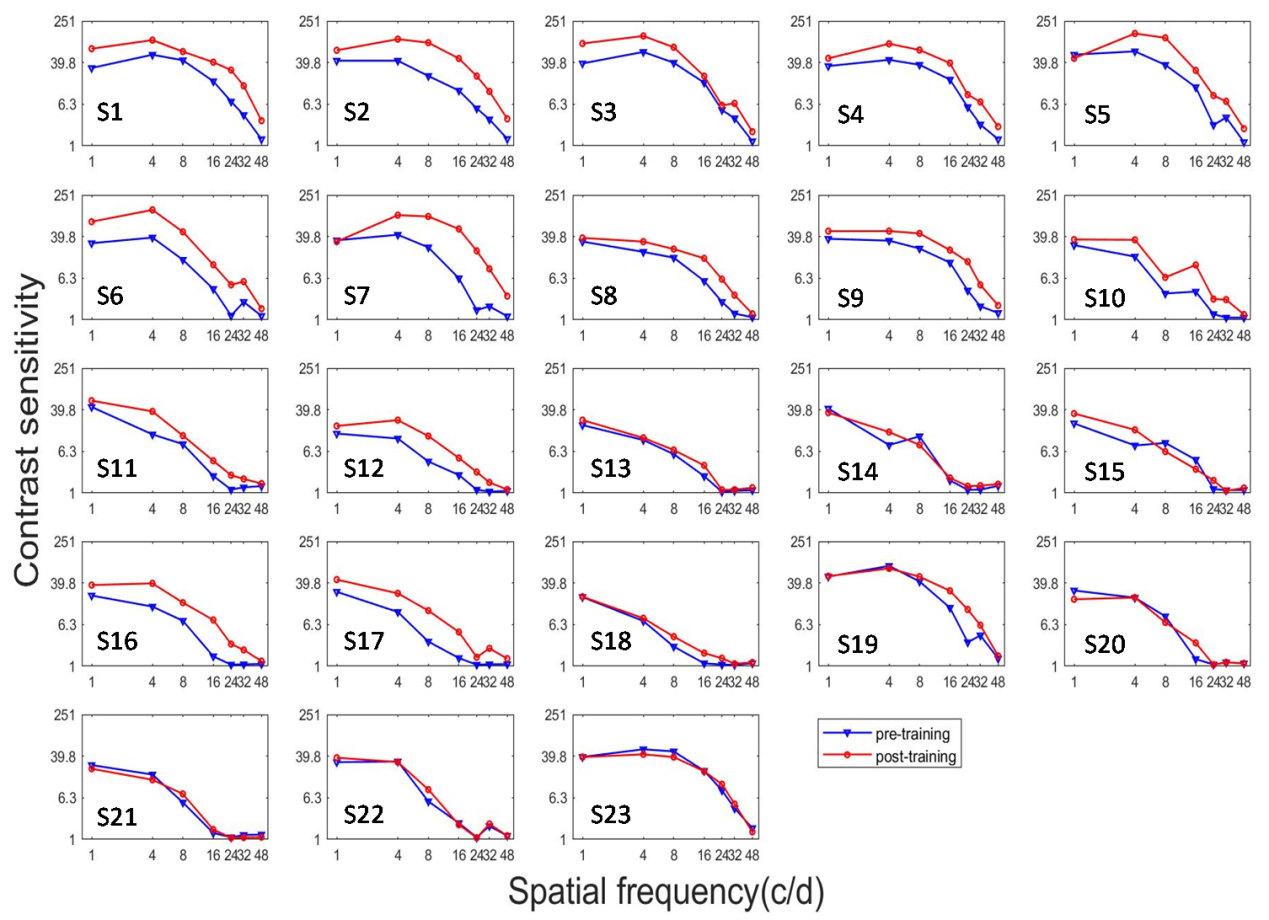
**

**FIGURE S1︱**Individuals’ contrast sensitivity functions (CSF) before and after training. The blue curves denote data in pretraining tests; red curves denote data in posttraining tests. Each figure represents an observer.


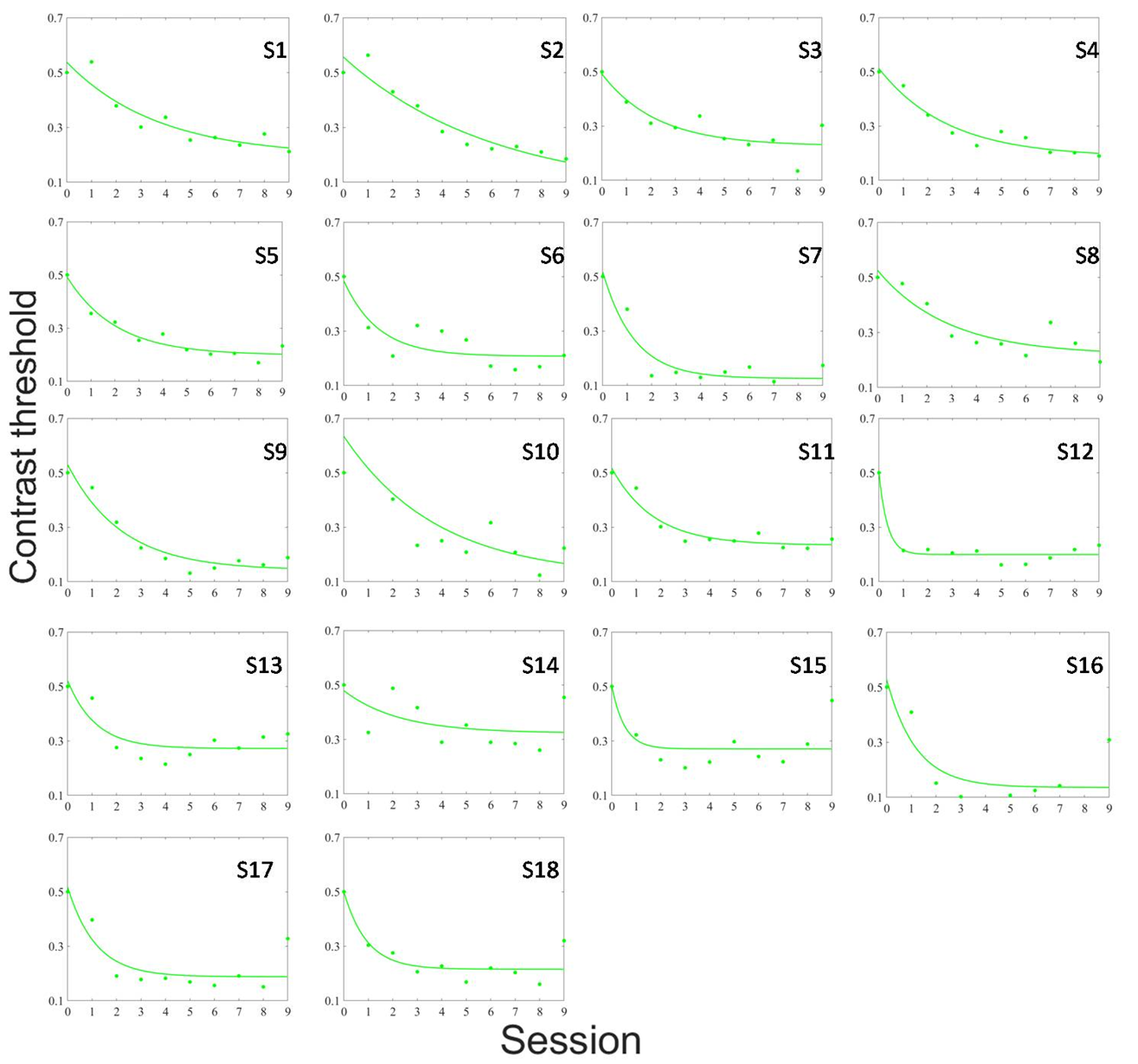


**FIGURE S2︱**The learning curves for each observer.
